# Paraurethral Leiomyoma as an Incidental Finding in Patient with Fibroid Uterus

**DOI:** 10.1155/2018/7042960

**Published:** 2018-02-07

**Authors:** Dmitry Fridman, Marnie Abeshouse, Alexander Sankin

**Affiliations:** ^1^Duke Medical Center, Department of Obstetrics and Gynecology, Durham, NC, USA; ^2^Sidney Kimmel Medical College, Thomas Jefferson University, Philadelphia, PA, USA; ^3^Department of Urology, Montefiore Medical Center, 1250 Waters Place, Tower One, Suite 706, Bronx, NY 10461, USA

## Abstract

Paraurethral leiomyomas are rare benign fibromuscular tumors developing from urethra. The presenting symptoms are usually related to mass effect. We present a case of an incidental diagnosis of a paraurethral leiomyoma in a patient with a fibroid uterus. Case was managed by hysterectomy concurrent with periurethral leiomyoma excision. Patient had uncomplicated clinical course. Due to close localization of paraurethral leiomyoma to urethra and bladder care must be taken to minimize the injury during resection.

## 1. Introduction

Paraurethral leiomyomas are rare and comprise approximately 5% of all paraurethral masses, which are present in 1 : 1000 women [1]. They arise from smooth muscle of the urethra and usually present with symptoms of pelvic pressure, urinary discomfort, and a protruding mass [2]. Here we present a case of an incidental diagnosis of a paraurethral leiomyoma in a patient with a fibroid uterus.

## 2. Case Report

**Figure 1:**
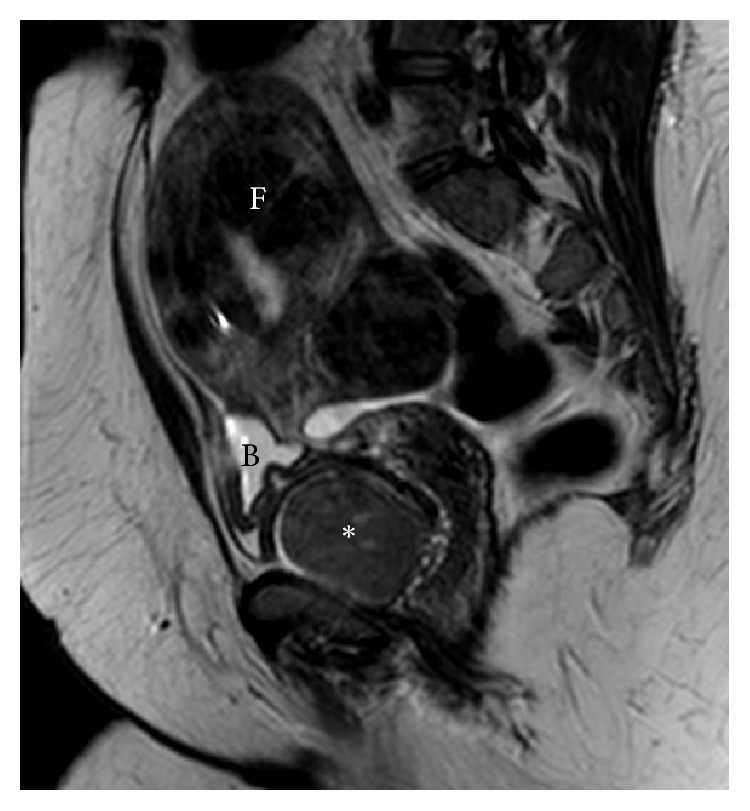
Preoperative imaging: T2-weighted pelvic MRI. F: fibroid uterus. B: bladder. ^*∗*^Paraurethral leiomyoma.

A 51-year-old woman presented to our clinic complaining of heavy vaginal bleeding, pelvic pressure, bladder incontinence, and discomfort during intercourse. Her exam confirmed the presence of a fibroid uterus extending to the level of umbilicus. She had grade 2 obesity and well controlled diabetes. Otherwise, there were no additional medical problems and no significant surgical history. After counseling, she decided to proceed with uterine artery embolization (UAE) and was referred to an interventional radiologist. A MRI was performed and an incidental finding was noted of an indeterminate 5.5 cm solid enhancing mass inferior to the bladder and inseparable from the superior anterior wall of urethra (Figure 1). Retrospectively reviewed with the collapsed bladder, it was difficult to differentiate the paraurethral mass from the mass effect of the fibroid uterus.

**Figure 2:**
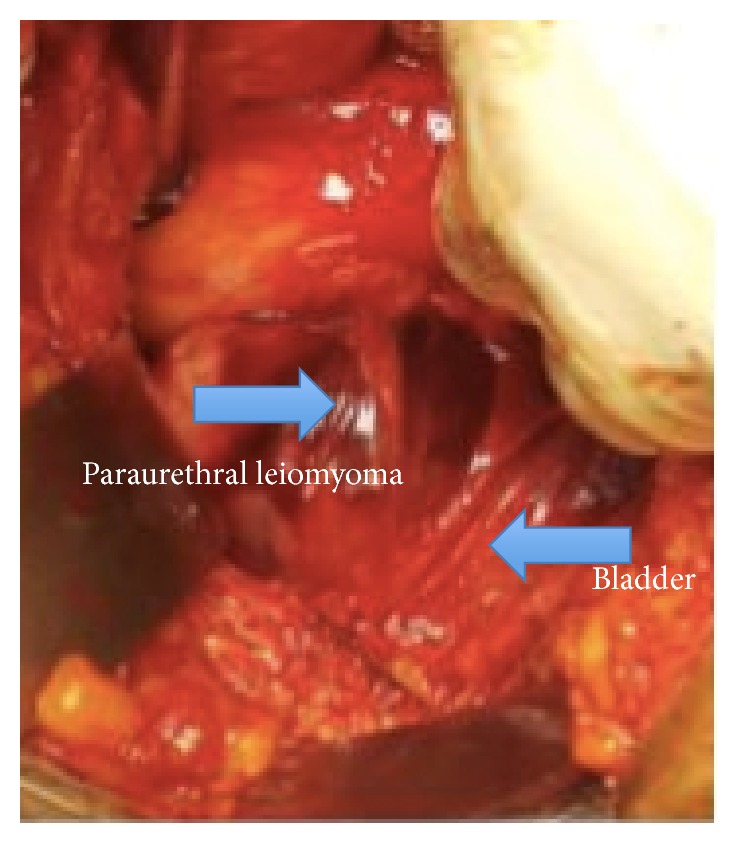
Intraoperative images of surgical field.

**Figure 3:**
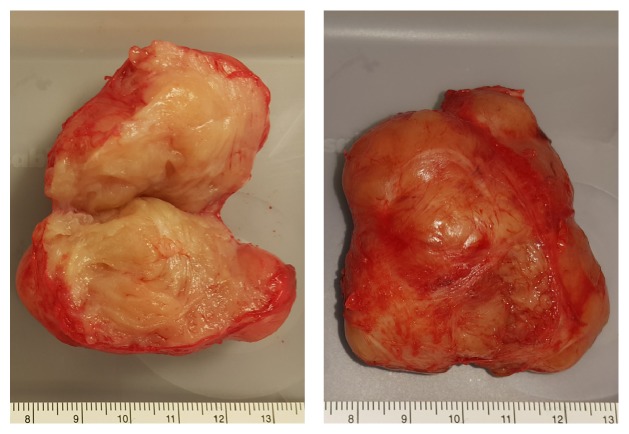
Gross image of excised paraurethral mass.

The patient then underwent cystoscopy, which ruled out invasion of the mass into the urethra, and an IR-guided biopsy of the mass, which returned spindle cell tumor with smooth muscle consistent with leiomyoma. Given these findings, additional counseling was provided and the patient elected to proceed with a hysterectomy and paraurethral mass resection. Abdominal access via the vertical midline laparotomy was chosen. Due to the size and position of paraurethral mass, we were concerned about injury to the trigone and therefore a prophylactic bilateral ureteral stenting was done in the beginning of the case. Uneventful total hysterectomy was performed. Entry into the retropubic space (Retzius) was achieved and a nodular mass was identified emanating from the anterior bladder wall. The superior and lateral attachments of this mass were easily dissected free; however the mass was densely adherent to the detrusor muscle (Figure 2). Sharp and blunt dissection was done to separate the mass from detrusor fibers. The detrusor layer of the anterior bladder wall was then reinforced with interrupted, absorbable sutures, and bladder instillation was done to confirm integrity. One week following the procedure, the patient had a Foley catheter removed and otherwise had an uneventful postoperative course. Paraurethral mass pathology returned as leiomyoma with negative surgical margins (Figure 3).

## 3. Discussion

Leiomyomas are benign tumors that develop from any smooth muscle, including the uterus and urethra. While uterine leiomyomas are common, paraurethral leiomyomas are rare and can occur in both men and women. However, they are three times more prevalent in women and, similar to uterine leiomyomas, are considered estrogen-dependent tumors that enlarge during pregnancy and regress during the postpartum period [3, 4]. Moreover, we found only a few case reports that describe coexistence of these pathologies. Although the average age of patients presenting with a paraurethral leiomyoma is 39.8 years [5], somewhat younger than average age of patients with a fibroid uterus, they have also been found in older women, such as our patient [6].

The presenting symptoms of patients with paraurethral myoma are usually related to urinary tract infection, mass effect, or dyspareunia [7]. Differential diagnosis typically comprises urethral diverticulum (84%), leiomyoma (7%), vaginal wall inclusion cyst (6%), Skene's gland cyst or abscess, urethral prolapse, urethral caruncle, and primary neoplasm (squamous cell carcinoma, urothelial carcinoma, and adenocarcinoma). Presentation may resemble other periurethral masses, and, as a result, a careful history and physical examination is crucial for the proper diagnosis [8].

These masses may develop along any part of the urethra; however, the majority of paraurethral leiomyomas present in the proximal segment. As a result, diagnosis via physical examination is typically standard protocol and is based on palpation of the vaginal mass, which, in our case, was complicated by presence of uterine fibroids. In addition, confirmation of diagnosis is encouraged and obtained through imaging, such as ultrasound or MRI [9]. Paraurethral leiomyomas typically appear as smooth, round, and solid ones, with size spanning approximately 1–8 cm [1].

If symptomatic, management usually involves local resection, which can be achieved both vaginally or abdominally depending on size, location, and comfort level of operating surgeon with the aim of minimizing recurrence and relieving symptoms. Due to the intimate relationships of the paraurethral mass with the urethra and, in our case, the bladder, care must be exercised so that injury to nearby organs and complications such as stress urinary incontinence and urethral stenosis are curtailed.
